# Complete genome sequence of a tetracycline-resistant bacterium, *Variovorax endophyticus* HR3-A, isolated from riverine water in Japan

**DOI:** 10.1128/mra.00743-26

**Published:** 2026-08-07

**Authors:** Miku Hayakawa, Takeshi Shoda, Tomoyasu Nishizawa

**Affiliations:** 1 Graduate School of Agriculture, Ibaraki University 12819, Ami, Ibaraki, Japan; 2 United Graduate School of Agricultural Science, Tokyo University of Agriculture and Technology 13125, Fuchu, Tokyo, Japan; 3 Green-Bio Technology Research Center, Ibaraki University 12819, Ami, Ibaraki, Japan; DOE Joint Genome Institute, Berkeley, California, USA

**Keywords:** antibiotic resistance, environmental microbiology

## Abstract

The genome sequence of *Variovorax endophyticus* HR3-A, a tetracycline-resistant bacterium that inhabits riverine water in Japan, was determined to identify the genetic characteristics associated with abilities involved in antimicrobial resistance.

## ANNOUNCEMENT

The impact of anthropogenic contaminants expelled into the environment through human activities such as domestic, industrial, and agricultural practices on antibiotic resistance has attracted widespread attention from the perspective of potential health risks (1, 2). To further investigate aquatic antibiotic-resistant bacteria, we isolated and sequenced the genome of a new aquatic *Variovorax* strain, HR3-A.

HR3-A was obtained from riverine water sampled on 21 June 2023, from Hokota River, Ibaraki Prefecture, Japan (36°11'29"N 140°31'52"E) (2). Genomic DNA was extracted from the colonies of HR3-A cells grown on R2A (FUJIFILM Wako Pure Chemical Corporation, Japan)—1.5% agar at 30°C for 3 days using the phenol-chloroform extraction method with the lysozyme buffer (2–4). Nanopore sequencing library using the HR3-A genomic DNA was prepared using a Native Barcoding Kit (SQK-RBK114.24; Oxford Nanopore Technologies, UK) and sequenced on a PromethION platform (R10.4.1, ONT). Base calling and adapter sequence removal were performed using Porechop v0.2.4 with Dorado v7.3 (SUP model) (5). Subsequently, cleaning of reads shorter than 1,000 bp was carried out using Chopper v0.7.0 (6). Finally, a total of 46,102 long reads (total base counts, 529,843,304; *N*_50_ value, 19,260 bp) were obtained from the Nanopore library. A 150-bp paired-end library using the same genomic DNA, which was fragmented using ME220 Focused-ultrasonicator (Covaris, USA), followed by purification and size selection using AMPureXP (Beckman Coulter, USA), was prepared using an MGIEasy PCR-free DNA library prep set (MGI Tech, China) and sequenced on the DNBSEQ-G400RS platform (MGI Tech). Adapter-sequence removal and read filtering were performed using Cutadapt v4.9 (7) (“--overlap 10,” “--minimum-length 51,” and “--quality-cutoff 20”) for MGI-reads. The filtered MGI reads were then downsampled to 2,000,000 paired-end reads (total base counts, 598,560,956) for subsequent analyses.

Nanopore reads were assembled using Trycycler v0.5.4 (8) to determine circular contigs, which were subsequently polished by Medaka v2.0.1. The Burrows-Wheeler Aligner v0.7.12 (9) and Pilon v1.23 (10) were used with the MGISEQ short-read data set. The genome sequences were annotated using DFAST v1.3.9 (11). The genome completeness was identified using CheckM v1.2.4 (12) within DFAST_QC v1.1.1 (13) using reference species identified based on the NCBI database. The completed genome was assessed according to average nucleotide identity (ANI) analysis within DFAST_QC as well. Default parameters were used for all software unless otherwise indicated.

The 10,011,889-bp genome (56.4× coverage) with a GC content of 69.0% was assembled into two contigs. The HR3-A genomic features are shown in Table 1. To determine the closest related species, whole-genome ANI was computed using draft genomic sequences of *Variovorax endophyticus* strain CY25R-8^T^ (NCBI accession number, GCF_025376855.1) (14), revealing 96.35% sequence similarity with the HR3-A genome. The HR3-A chromosome contains putative genes involved in the resistance of antibiotics (*bla*, ampicillin and *tetA*, tetracycline). We also found genes encoding conjugative type 4 pilus and putative endoglucanase associated with interactions among bacteria and plants.

**TABLE 1:** Genome features of *Variovorax endophyticus* HR3-A

Genome features	Contig 1	Contig 2
Total Length (bp)	7,570,958	2,440,931
Alias	Chromosome	Chromid
Form	Circular	Circular
GC content (%)	69.1	68.7
No. of CDSs	6,936	2,182
No. of rRNAs	6	3
No. of tRNAs	76	7
No. of tmRNAs	1	0
Completeness (%)	98.77	

## Data Availability

The genome sequences of HR3-A were deposited in DDBJ/ENA/NCBI under sequence read archive numbers DRR1058126 and DRR1058127 for the Nanopore and MGISEQ raw reads, respectively, and under accession numbers AP048872 and AP048873.
